# Draft genome sequencing of *Brucella melitensis* isolated from aborted Iraqi sheep

**DOI:** 10.1128/mra.01213-23

**Published:** 2024-03-15

**Authors:** Yahia I. Khudhair, Hayder Naji Ayyez, Rosa Estela Quiroz-Castañeda, Fernando Martínez-Ocampo, Mourad Ben Said

**Affiliations:** 1 Department of Internal and Preventive Medicine, College of Veterinary Medicine, University of Al Qadisiyah, Al Diwaniyah, Iraq; 2 Unit of Zoonotic Disease Research, College of Veterinary Medicine, University of Al-Qadisiyah, Al Diwaniyah, Iraq; 3 Laboratory of Anaplasmosis, National Center for Disciplinary Research in Animal Health and Safety, INIFAP, Jiutepec, Morelos, Mexico; 4 Postdoctoral Stay Program in Mexico 2022(3), Academic Modality-Initial, CONAHCYT, Mexico City, Mexico, Bioinformatics and Evolutionary Genomics Laboratory, Cellular Dynamics Research Center (CIDC) Morelos, Cuernavaca, Morelos, Mexico; 5 Laboratory of Microbiology, National School of Veterinary Medicine of Sidi Thabet, University of Manouba, Manouba, Tunisia; 6 Department of Basic Sciences, Higher Institute of Biotechnology of Sidi Thabet, University of Manouba, Manouba, Tunisia; Indiana University, Bloomington, Bloomington, Indiana, USA

**Keywords:** *Brucella melitensis*, brucellosis, aborted sheep, Iraq

## Abstract

Brucellosis remains a significant zoonotic disease, with *Brucella melitensis* maintaining endemicity in Middle Eastern nations. This study presents the draft genome sequencing of an Iraqi *B. melitensis* strain, representing a crucial step in monitoring virulence, antimicrobial resistance, and exploring the diversity and evolution of the *Brucella* genus.

## ANNOUNCEMENT

Brucellosis, an infectious disease caused by *Brucella* species, is typically transmitted to humans through the consumption of unpasteurized milk and dairy products, undercooked meat, or direct contact with livestock.

In this study, *Brucella* strains were successfully isolated from clinical samples obtained from the fetal stomach contents of sheep aborted fetuses and placental cotyledons in Al Qadisiyah, Iraq (31.90 N 44.50 E). Samples of aborted fetuses and placental tissues from suspect cases were cooled at 4°C immediately after being taken and then were transported to the laboratory of the Zoonotic Research Unit of College of Veterinary Medicine/Al-Qadisiyah University (Iraq) within 2 h. At the laboratory, Aborted fetuses were necropsized and specimen were taken from abomasum contents as follows: the abomasum wall was seared with a heated spatula; contents were obtained by plunging the tip of a sterile syringe through the seared area; and some of the contents were transferred. Directly, the abomasum contents were inoculated onto 7% sheep blood agar (Oxoid CM 271) plates and *Brucella*-enriched media (Oxoid CM 0169). All cultures were incubated at 37 C° with 5% CO_2_ for 5–7 days. *Brucella* identification and species differentiation were accomplished using PCR protocols (1).

DNA extraction from the specimens was obtained using a kit (Bacterial Genomic DNA kit; Geneaid, Taiwan), and these DNA samples were subjected to PCR analysis using forward primer 5′-GGCGTGTCTGCATTCAACG-3′, (151229–151211) and reverse primer 5′-GGCTTGTCTGCATTCAAGG-3′, (150390–150408) designed to detect *Brucella* spp. The resulting PCR products were sequenced by Sanger at Macrogen (Korea) to confirm the isolates’ identity. Pure bacterial colonies were selected and cultured in heart and brain infusion broth. The bacterial pellets were obtained by centrifugation at 16,000 rpm for 1 min. Then, DNA extraction was carried out using a kit (Bacterial Genomic DNA kit, Geneaid). The DNA concentration was assessed using Nanodrop (Thermo Fisher Scientific), yielding a concentration of 174 ng/µL in a final volume of 90 µL. Sequencing libraries were generated using TrueSeq Nano Library kit for Illumina (New England Biolabs), and the genome sequencing of *Brucella melitensis* strain Al Qadisiyah was performed using the Illumina NovaSeq platform with paired-end sequencing at Macrogen (Korea).

Illumina sequencing libraries were generated with the High Throughput Library Preparation Kit (TruSeq Nano DNA 350; Illumina, San Diego, CA, USA) according the manufacturer’s protocol. Whole-genome libraries were indexed with NEXTflex barcodes [Bio (SIC) Scientific, Austin, TX, USA]. Sequencing was performed using a NovaSeq X platform, with genomic DNA sequencing generating 22,810,912 paired-end reads with a length of 151 nucleotides for each read (Illumina). The Illumina adapter sequences were removed from the paired-end reads with the ILLUMINACLIP option of the Trimmomatic program (version 0.39) (2). Subsequently, nucleotides with a low Phred quality (*Q* < 13) were removed from the paired-end reads with the DynamicTrim algorithm of the SolexaQA ++package (version 3.1.7.3) (3). The quality of the original paired-end reads (obtained from genomic DNA sequencing), trimmed with the Trimmomatic and SolexaQA ++programs, was evaluated with the FastQC program (version 0.12.1) (4). The trimmed paired-end reads were used to *de novo* assemble the genome with the SPAdes program (version 3.15.4) (5) using k-mer values between 21 and 127. The assembled genome contigs, with a length of less than 500 bases and assembly coverage of less than 1.0, were removed with a Perl script. The taxonomy of each contig of the assembled genome was identified with the Kraken program (version 2) (6). The genome quality and characteristics of *B. melitensis* strain Al Qadisiyah were evaluated with the CheckM program (version 1.2.2) and the QUAST program (version 5.0.2), respectively (7). The first genome assembly generated 65 contigs. The Kraken program identified 20 contigs specific for *B. melitensis* and 45 contigs belonging to other organisms. The draft genome of the 20 contigs of *B. melitensis* has a total length of 3,290,241 bp. The longest contig is 610,126 bp, a GC content of 57.24%, an N50 length of 299,339 bp, an L50 value of 4, and a depth of coverage of 284.9×.

Analysis of the CheckM program shows a percentage of “completeness” and “contamination” of 99.38% and 0.52%, respectively. This analysis was performed for the 20 contigs of the draft genome. The chromosomes possess 3,318 coding sequences, 3 rRNAs, and 51 tRNAs genes.

Additionally, the first annotation we performed was in Prokka software (8). However, when the genome was submitted to GenBank, this information was unintentionally not included. Therefore, the National Center for Biotechnology Information (NCBI) annotated the genome with software Prokaryotic Genome Annotation Pipeline (https://www.ncbi.nlm.nih.gov/datasets/gene/GCF_034296205.1/).

The accession numbers for both the assembly and raw reads are GCA_034296205.1 and SRR27129338, respectively.

## Data Availability

This Whole Genome Shotgun project has been deposited at DDBJ/ENA/GenBank under accession number JAXKQI000000000. The version described in this paper is version JAXKQI010000000. Sequence Read Archive accession number is SRR27129338.
